# Movement, residency, and behavioral plasticity of reef manta rays in the Samarai Islands of Papua New Guinea

**DOI:** 10.1371/journal.pone.0344615

**Published:** 2026-05-28

**Authors:** Anna M. Knochel, Joanna L. Harris, Shannon E. Murphy, Annie Murray, Guy M.W. Stevens, Mark V. Erdmann

**Affiliations:** 1 The University of the Sunshine Coast, Sippy Downs, Australia; 2 The Manta Trust, Corscombe, Dorset, United Kingdom; 3 University of Plymouth, Plymouth, United Kingdom; 4 Columbia University, New York, New York, United States of America; 5 Conservation International Asia-Pacific Field Division, Auckland, New Zealand; 6 Re:wild, Austin, Texas, United States of America; Fisheries and Oceans Canada, CANADA

## Abstract

The reef manta ray (*Mobula alfredi*) is a highly mobile pelagic marine ray found throughout the tropical and subtropical waters of the Indo-Pacific, but investigation into their behavior and ecology within Papua New Guinea has not been previously undertaken. Furthermore, the home range, dispersal characteristics, and inter-seasonal fine-scale habitat use of this species is limited. To address these data gaps and investigate the vertical and horizontal habitat use of a previously unstudied population, SPLASH10-F-321A pop-off archival satellite tags were used to track ten adult individuals from 4–181 days between 2016–2018 across two distinct monsoonal periods in the Samarai Islands of Milne Bay, southeastern Papua New Guinea. Our findings indicate strong site-attached movement patterns for reef manta rays in this region, with 75% of relocations occurring within ten kilometers of the tagging site. While occasional movements beyond this range were observed, the maximum displacement distance was 86.9 km, and no consistent seasonal differences in horizontal displacement distance were detected. Tagged rays displayed a clear preference for the Samarai Islands and the Papuan Plateau across both monsoons, with shallow bathymetry and elevated chlorophyll-a values driving observed habitat preferences. We found evidence for shifts in vertical occupancy of the water column that corresponded with the mixed layer depth; dives were deeper when the mixed layer depth was shallow, suggesting that reef manta rays can exhibit behaviorally plastic responses to seasonal variations in oceanographic conditions. These findings provide the first insight into the movement ecology of this reef manta ray population that can be used to inform the development of economically valuable manta ray tourism practices and a sustainable management plan in the region.

## Introduction

The reef manta ray (*Mobula alfredi*) is one of ten recognized species in the monogeneric Mobulidae family [1–4]. It is a conspicuously large pelagic zooplanktivore commonly found in neritic habitats and island archipelagos throughout its tropical and subtropical range in the Indian and Pacific Oceans [2,5]. While reef manta rays are not considered keystone species or apex predators, they possess several traits that are functionally important in the oligotrophic tropical and subtropical systems where they are found. The species is a forager that targets low trophic levels and exhibit aggregation behaviors that are strongly linked to regional or local oceanography [6,7], underscoring its potential role as an ecosystem sentinel [8]. Reef manta rays also utilize deepwater habitats to at least 711 m and derive some of their diets from deep-water sources, creating a link between mesopelagic and shallow reef habitats [9–12].

Reef manta rays are commonly found in areas overlapping human presence and the predictable seasonal occurrence of large-scale aggregations in some regions has been instrumental in developing sustainable ecotourism operations that bolster local economies [13]. However, reef manta rays are threatened by a range of sublethal anthropogenic impacts, such as fishing line entanglement, vessel strikes, and irresponsible tourism, and lethal impacts, such as directed fishing and bycatch [13–17]. The management and conservation of reef manta rays is further complicated by the trade of their gill plates, a highly valued body part that is deceptively marketed for medicinal use [18]. Furthermore, the species lives in small geographically isolated populations throughout its range and is one of the least fecund of all elasmobranchs with one of the lowest intrinsic rates of population increase [19]. Declining populations and the continued persistence of sublethal and lethal threats are causes for concern and the reef manta ray is now listed as Vulnerable to Extinction on the IUCN’s Red List of Threatened Species [20–22]. Therefore, there is an urgent need for a detailed understanding of the spatial and temporal dynamics of *M. alfredi* habitat utilization to inform and develop regionally suitable protection strategies.

Papua New Guinea is one of the most biodiverse regions on earth and is home to critical habitats for many threatened and endangered elasmobranch species [23]. Through a combination of local indigenous knowledge and exploratory liveaboard dive trips, a conspicuous but undescribed population of reef manta rays was identified in the Samarai Islands of Milne Bay Province of the southeastern part of the country (Rob Vanderloos personal communication). However, the current lack of baseline knowledge of their home range and habitat use raises concerns about potential anthropogenic threats in the region. For example, across seven trips with fishery observers, one reef manta ray was captured as bycatch in the Gulf of Papua Prawn Trawl fishery [24]. Although this area lies ~650 km to the west of the Samarai Islands, reef manta rays are capable of swimming these distances, as demonstrated by the movements of reef manta rays along connected continental shelves [25,26]. Much closer to home, four decapitated reef manta ray heads were observed strewn on the bottom, amongst seven finned shark carcasses, in the Dumoulin Islands (30–40 km south of the Samarai Islands) during a liveaboard survey in May 2016 (Erdmann, pers. obs.). Furthermore, a major shipping route lies ~100 km to the east of the Samarai Islands, which may overlap with this population’s range and possibly result in negative interactions [27]. Given the variation in broad-scale movement patterns and residency of reef manta populations and the potential for this area’s development as a new ecotourism destination, a targeted investigation of this reef manta ray population’s movement characteristics is urgently required. Here, we deployed a suite of pop-up satellite archival tags equipped with GPS capabilities to 1) delineate the spatial boundaries of this aggregation and understand its dispersal characteristics, 2) identify important habitats for reef manta rays of the Samarai Islands and surrounding area, 3) assess seasonal shifts in vertical and horizontal movements, and 4) determine the oceanographic factors contributing to reef manta ray habitat use.

## Methods

### Ethics

Permission was received by local reef owners and communities to dive in Milne Bay Province and deploy eleven SPLASH10, Fastloc GPS-enabled towed tags on reef manta rays (Wildlife Computers Inc, Redmond, WA, USA). Research was approved by the Papua New Guinea National Research Institute and Papua New Guinea Immigration and Citizen Services (VARN #69990471946), and by Columbia University [The Institutional Animal Care and Use Committee (IACUC) approval #AAAT8451].

### Study location, design and data collection

**Fig 1:**
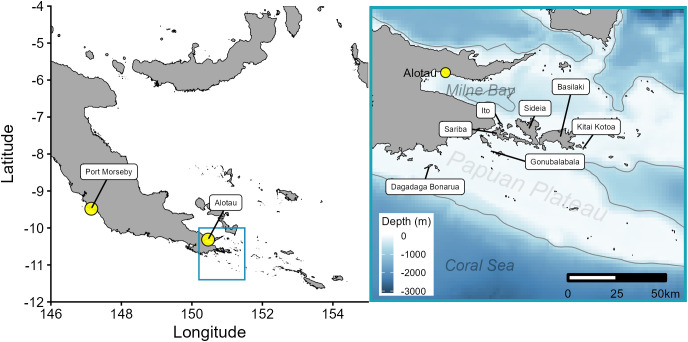
Map of the study area. Eastern Papua New Guinea (left panel) and Milne Bay (right panel). The light grey line represents the 500 m isobath [38,39]. National boundaries were sourced from the Papua New Guinea National Statistics Office [34].

Research was conducted in Milne Bay Province, Papua New Guinea (Fig 1), at two reef manta ray cleaning sites: Gonubalabala Island (10.685782° S, 150.678461° E) and “Manta Sideia Heaven” off Sideia Island (10.540661° S, 150.860138° E). These sites are part of the Samarai Islands, located south of Milne Bay, where the three largest islands—Sariba, Sideia, and Basilaki—are separated by south-north aligned channels. Importantly, the second cleaning station site at Sideia Island was only identified after the results of the initial round of tagging at Gonubalabala, which revealed that tagged manta rays were aggregating in the northern channel between Sideia and Sariba Islands. A subsequent survey at this site confirmed both the presence of an active cleaning station as well as a feeding aggregation.

Three research expeditions were conducted to deploy SPLASH10-F-321A satellite tags (Wildlife Computers Inc, Redmond, WA, USA) on reef manta rays to capture behavioral patterns throughout the year. Two tags were deployed at Gonubalabala Island in May 2016. Six additional tags were deployed at “Manta Heaven” off Sideia Island in December 2016, followed by three more at the same site in May 2018. These pop-off archival tags are equipped with a Fastloc GPS, which allows locations to be obtained even when the tag only surfaces briefly (0.2 seconds). In addition, location data were obtained through the Argos satellite system, which estimates positions using the Doppler shift of transmissions emitted by the tag during satellite overpasses. The accuracy of Argos positions is influenced by the number of transmissions received and the processing method (least squares or Kalman filtering), with each point assigned one of seven location quality classes (LCs) [28]. Higher quality locations (LC = 0–3) are derived from four or more transmissions and are accompanied by an estimated error radius, which reflects the expected spatial uncertainty of the position [28].

All tags were programmed to assign depth and temperature into discrete bins to facilitate data compression and transmission via the Argos satellite system, and to enable standardized comparisons across individuals. Depths were grouped into nine bins (0 m, 0–1 m, 1–5 m, 5–50 m, 50–100 m, 100–200 m, 200–300 m, 300–400 m, 400–500 m) while temperatures were assigned to 14 bins (5–10 °C, 10–15 °C, 15–20 °C, 20–22 °C, 22–24 °C, 24–25 °C, 25–26 °C, 26–27 °C, 27–28 °C, 28–29 °C, 29–30 °C, 30–31 °C, 31–32 °C and 32–33 °C). These data were summarized into 12-hour periods to investigate potential diel differences in activity, corresponding with daytime (6:00 am to 6:00 pm UTC + 10) and nighttime (6:00 pm to 6:00 am UTC + 10). During subsequent data analyses and due to the minimal time spent in some bins, the proportion of time spent in the bins between 0–5 m (n = 3) and between 100–500 m (n = 4) were summed. Tags were programmed to detach from the manta ray after 180 days of deployment. Once detached from the animal, the tags floated on the surface and transmitted data to the Argos satellite system until the battery expired.

All tags were deployed using a 2m pole spear (“Hawaiian sling”) on freely swimming individuals using a titanium anchor (64 L x 16 W x 1 mm) and stainless-steel tether attachment with lengths between 25 cm and 30 cm. Tags were positioned on the anterior dorsal surface, ensuring the anchor did not penetrate the body cavity and the tag did not extend beyond the posterior edge of the pectoral fin. Prior to tag deployment, each individual reef manta ray was photographed ventrally to document its unique spot pattern, enabling individual identification based on established photo-identification techniques [29]. Sex and visually estimated size (disc width, DW) were also recorded, with sex determined by the presence or absence of claspers. Maturity was assessed using estimated size (DW) at sexual maturity: 280 cm for males for males and 320–340 cm for females [30,31].

### Data analysis and visualization

All analyses were conducted in R version 4.2.3 [32].

Based on the GPS and Argos data, it was determined that some tags were shed prematurely from the manta rays and drifted away from the region. When a tag is floating at the surface, the likelihood of achieving high-quality LCs increases substantially due to the greater number of successful transmissions. To identify potential shedding events, we manually assessed each track and determined the likely date of tag shedding based on the frequency and quality of Argos positions [33]. The deployment end date was determined to be the last likely Fastloc GPS or Argos point in the track. We elected not to remove Argos and GPS-derived points if they were over land due to the complex regional topography and number of islands in the study area. Instead, positions that fell over land were snapped to the nearest land-ocean boundary as defined by a shapefile of administrative boundaries for Papua New Guinea [34].

Southeastern Papua New Guinea experiences a monsoonal climate system dominated by the Australasian monsoon, which includes a northwest (NW) monsoon (November – April), characterized by weak variable winds, and the southeast (SE) monsoon (May – October), dominated by southeasterly winds [35,36]. To visualize these patterns throughout the year and identify potential variables driving observed movements in tagged reef manta rays, the monthly averaged u (eastward) and v (northward) components of wind were extracted from Copernicus Marine Service’s Climate Data Store (https://cds.climate.copernicus.eu/datasets) at 0.25 x 0.25 degrees resolution and averaged across an extent that covered the general region (150.1 °E, 151.5 °E, 11.2 °S, 10.4 °S). Sea surface temperature (SST) data were extracted from the same platform at the same resolution as previous. As a proxy for surface ocean productivity, chlorophyll-a (mg/m^3^) data at 4 km resolution was downloaded from the Ocean Color Database (https://oceancolor.gsfc.nasa.gov/). Monthly vertical temperature profile models were extracted from Copernicus Marine Service’s Climate Data Store at the closest available data point to the study area (11.91667 °S, 150.4167 °E) and the mixed layer depth (MLD) was determined to be the depth at which the density increased by 1°C relative to 10 m below the surface [37]. Additionally, bathymetry data was sourced by querying the ETOPO global relief 2022 database hosted by NOAA using the ‘marmap’ package [38,39].

Argos and Fastloc position estimates were used to calculate displacement (distance from the tagging location to each recorded point), stepwise distances between successive points, and movement speeds for each track. Mean displacement from the tagging location was then computed across all tags. A cutoff speed of 20 km/hr was applied to identify and exclude improbable locations based on our understanding of the species’ swimming abilities; however, none of the recorded positions exceeded this threshold, so no data points were removed [27]. Due to their widespread use in the field of movement ecology for several decades and therefore their utility in comparing home ranges between studies [40–42], minimum convex polygons (MCPs) were calculated in the ‘adehabitatHR’ package for each individual using the raw Argos and Fastloc positions and collectively for the tagged cohort by pooling all positions. Since Argos and Fastloc positions are only obtained when the animal is at the surface – and therefore are a fraction of the true number of locations that this species utilizes within its habitat – 100% of points were considered in calculating the MCPs (typically this is set at 95%).

Fastloc and Argos position estimates were highly irregular and directed into a state-space model (SSM) with a simulated correlated random walk using the ‘aniMotum’ package to interpolate the track by providing positions every 12 hours and determining where animals may have moved between surface points [43]. Parts of individual tracks where the animal was not detected for more than seven days were segmented to avoid inaccurate interpolations across large temporal gaps [44]. Furthermore, only tracks with 10 or more observations were kept, which resulted in the removal of three individuals from the SSM track outputs (IDs 157297, 158697, and 174991). The resulting standardized tracks were analyzed using the ‘fit_mpm’ function to calculate a move persistence index known as ‘g’ that ranges between 0–1. The move persistence index estimates likely behaviors between points, with low index numbers indicating area restricted searching and high index numbers indicating transience. Kernel utilization distributions (KUDs) were calculated using the SSM outputs with the package ‘adehabitatHR’ and an href smoothing parameter to determine 50% core activity space and 95% home ranges for the collective tagged cohort of reef manta rays (n = 7). We used the resulting SSM estimated locations instead of Fastloc and Argos position estimates to establish KUDs to avoid biasing distributions according to surfacing behavior and to incorporate estimated movement tracks between points of surfacing.

To determine if manta ray movements inferred by the SSM were associated with environmental factors, we used boosted regression trees to model the effects of satellite-derived environmental variables and bathymetry on SSM positions. Boosted regression trees (BRT) repeatedly and randomly sample a specified fraction of the data and fit decision trees in an iterative fashion, with subsequent trees built by adding more weight to previous, more highly erroneous predictions [45]. In this way, BRTs are equipped to deal with the serial autocorrelation that arises in tracking data. We created a set of pseudoabsences that temporally matched the SSM position and whichwere randomly generated from a buffer zone of 21 km, the largest distance moved by a tagged animal within 12 hours to ensure biological plausibility [46]. After extracting variable data (bathymetry, SST, chlorophyll-a) from coordinates for both presence and absence points, we calculated pairwise correlation coefficients and variance inflation factor (VIF) estimates [47] to ensure they were in an acceptable range where noninformative predictors were not hindering model performance [coefficients <0.6 and/or VIF estimates <3.5 [7,47]].

A series of BRT models with a Bernoulli distribution (presence and pseudo-absence) were fit using the ‘gbm.step’ function from the ‘dismo’ package. Different models were iteratively built increasing tree complexity (tc = 1–3) and learning rates (lr = 0.01–0.001), bag fraction (bf = 0.5 and 0.7) and step size (ss = 25 and 50), resulting in 36 models (S1 Table). Ten-fold cross-validation procedure was employed to evaluate performance, during which the model is fitted to training data and then is tested against a withheld portion of the dataset. To assess model performance, the area under the receiver operating characteristic curve (AUC) was calculated for both the training (_T_AUC) and withheld portion of the dataset (cross-validation AUC, _CV_AUC), following methods outlined by Dedman et al. (2017), Froeschke et al. (2010), and Elith & Leathwick (2017) [48–50]. AUC values were interpreted according to standard thresholds: < 0.5 (fail), 0.6–0.7 (poor), 0.7–0.8 (acceptable), 0.8–0.9 (excellent), and >0.9 (outstanding) [51]. The percentage of deviance explained by the model was determined using the pseudo determination coefficient (*D*^*2*^), calculated using the following form [52]:


$$\begin{array}{c} D^{2}=\;1-\frac{Residual\;Deviance}{Total\;Deviance} \end{array}$$

The difference between the _T_AUC and _CV_AUC (ΔAUC) serves as an indicator of potential overfitting in the model, with smaller ΔAUC values suggesting less overfitting [48]. Optimal model performance is reflected by high AUC scores for both _T_AUC and _CV_AUC alongside a minimal ΔAUC [48]. The final model was fitted with tc = 3, lr = 0.01, bf = 0.7, and ss = 25 (S1 Table). Partial dependency plots were generated with confidence intervals (95%) obtained from 1,000 bootstrap replicates of the final model [7,47].

Vertical behavioral data was available in two forms: histograms of summarized time spent in predetermined bins over 12-hour periods corresponding to day and nighttime periods and maximum diving depths reached within the same 12-hour period. Histogram data of diving behavior was pooled across individuals and analyzed using PERMANOVA to see if time spent in depth bins was affected by the time of day, month, and interaction of these two terms. These data were ordinated and visualized using non-parametric dimensional scaling (NMDS) and additionally represented in a heat map to visualize where and when shifts in occupancy occurred across monsoons.

Finally, we applied generalized linear mixed models (GLMMs) and a generalized additive mixed model (GAMM) with a Gamma error distribution and a log link function using the “glmmTMB” and “mgcv” packages to examine differences in distances moved and depth use between monsoons and any interaction between maximum dive depth and mixed layer depth [53,54]. This modeling approach accommodates the complex structure of the dataset: track lengths varied substantially across individuals, resulting in uneven sampling effort and non-normal data distributions. GLMMs are well suited for this kind of ecological data, as they allow for non-Gaussian error distributions and can accommodate unbalanced sampling designs. Furthermore, GAMMs extend GLMMs by allowing for data-driven estimation of flexible, nonlinear relationships through the application of smoothing splines to predictor variables. This capability is often critical for accurately representing the nuanced patterns inherent in ecological datasets. Manta ID was included as a random effect to account for repeated observations within individuals and to control for inter-individual variability.

Specifically, to determine whether the magnitude of manta ray relocations differed between monsoons, we fit a GLMM using distance as the dependent variable and monsoon as a categorical fixed effect. Similarly, GLMMs were used to assess whether the proportion of time spent above 5 m and between 50–100 m differed significantly between monsoons. To investigate how diving behavior varied with shifting oceanographic conditions, maximum diving depths were temporally paired with the monthly mean MLD, and a GAMM was fitted using this pooled dataset. For all GLMMs and the GAMM, model selection was conducted by comparing each candidate model to a null intercept-only model using corrected Akaike information criterion (AICc) and AICc weights. The model with the lowest score, differentiated by more than 2 AICc units, was considered the best-supported model [55]. For models differentiated by less than 2 AICc units, the most parsimonious model was chosen.

Model diagnostics were evaluated using the DHARMa package [56]. The GLMM evaluating the relationship between time spent in the < 5 m depth bin showed good model fit with residuals following expected distributions and no evidence of overdispersion or heteroscedasticity. For the GAMM examining the relationship between mixed layer depth and maximum dive depth, the Kolmogorov-Smirnov test and combined adjusted quantile test indicated deviations from expected uniform residual distributions. These deviations might result from the sensitivity of these tests to large sample sizes (n = 775). However, visual inspection of QQ plots showed residuals followed the expected diagonal pattern, and dispersion tests were nonsignificant. To provide robust uncertainty estimates, we generated bootstrapped 95% confidence intervals by resampling the full dataset with replacement 500 times, refitting the model to each bootstrap sample, and computing percentile-based confidence intervals from the resulting prediction distributions.

## Results

### Monsoonal classification

**Fig 2:**
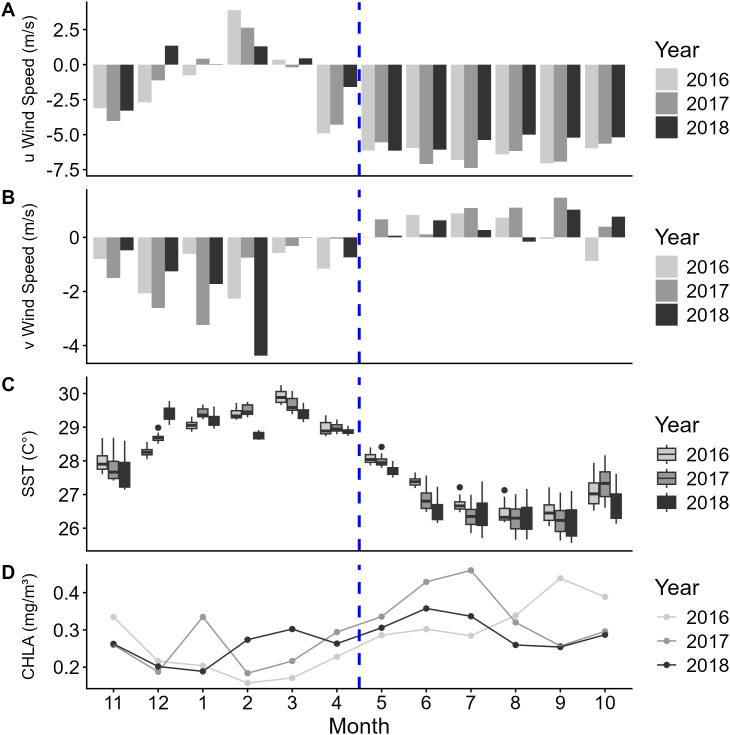
Seasonal environmental conditions in the study area. Visual of mean monthly observed (A) eastward (u) and (B) northward (v) components of wind in the study area, (C) monthly boxplots of sea surface temperatures (SST; °C) and (D) mean chlorophyll-a (CHLA; mg/m^3^) during the study period, 2016–2018. The dashed blue line represents the point of transition between the NW and SE Monsoons.

Satellite derived sea surface temperature, chlorophyll-a values, and wind strength data support the classification of the NW and SE Monsoons in the Milne Bay Province, with two-sample t-tests performed on monthly means showing significant differences between the monsoons for all four variables (SST: t = 5.20, df = 9.37, p < 0.001; chlorophyll-a: t = −4.96, df = 32.71, p < 0.001; v wind: t  = −4.78, df = 21.12, p < 0.001; u wind: t  = 8.16, df = 18.87, p < 0.001). Based on the average observed speeds of both eastward (u) and northward (v) components of wind, November – April were categorized as the Northwesterly (NW) Monsoon and May – October as the Southeasterly (SE) Monsoon (Fig 2). However, the beginning and end of the NW Monsoon (November and April) could be described as transition periods between the SE and NW Monsoons. Sea surface temperature increased during the NW Monsoon and decreased in the SE Monsoon (Figs 2 and S1 Fig). Regionally, sea surface temperatures in the Samarai Islands and Conflict Islands remained on average 1 °C lower than the surrounding region during the NW Monsoon (S1 Fig). Chlorophyll-a values increased across the region during the SE Monsoon compared to the NW Monsoon, although pockets of high productivity were evident in some locations in the NW Monsoon (Figs 2 and S2 Fig). The MLD gradually deepens during the SE Monsoon, reaching peak depth in August and September before shallowing during the NW Monsoon (S3 Fig).

### Horizontal distribution

Tracks ranged from 4–181 days and provided 566 locations that consisted of ten deployment points, 383 Fastloc GPS positions and 173 Argos positions (LC = 0–3, A and B). On average, individuals recorded a position every 51.7 hours. No tags were physically recovered, and one did not report for unknown reasons. Only one of the reporting tags released on schedule 181 days post deployment (ID 162382) while all the others released prematurely (4–146 days), resulting in an overall mean retention rate of 76.7 ± 51.2 days (Table 1). A total of five and three animals were only tracked during the SE and NW Monsoons, respectively, while the last two animals (IDs 157298 and 162382) with the longest tracking periods recorded data during both monsoons (S4 Fig). However, only one position estimate occurred in the SE monsoon for ID 157298. Nearly all tagged animals that successfully transmitted data were all considered mature based on their estimated size, apart from one female with an estimated disc width of 3m.

**Table 1:** Summary of tagging and tracking metrics for reef manta rays.

Tag ID	Start Date	End Date	Track Period	Mons.	% Dec.	Sex	DW (m)	Maturity	# Positions	Max. Disp. (km)	Track Length (km)	MCP (km^2^)	Max Depth
152724	2016-May-25	2016-Aug-19	86	SE	55	F	3.3	Adult	59	36.8	364.2	998	64
158697	2016-May-25	2016-May-29	4	SE	82	F	3.3	Adult	5	16.5	45.1	69	48
157295	2016-Dec-26	2017-Feb-17	53	NW	83	F	3.8	Adult	30	9.6	65.1	74	80
157296	2016-Dec-26	2017-Mar-11	75	NW	33	F	3.0	Unknown	68	14.8	172.3	152	64
157297	2016-Dec-19	2017-Feb-13	56	NW	91	F	3.8	Adult	13	47.3	107.8	313	336
157298	2016-Dec-19	2017-May-14	146	NW/SE	69	M	3.0	Adult	35	86.9	862	3,742	>400
162381	2016-Dec-19	NA	NA	NW	DNR	M	3.2	Adult	NA	NA	NA	NA	NA
162382	2016-Dec-19	2017-Jun-18	181	NW/SE	46	F	3.8	Adult	54 / 0	54.8	838.7	2,463	>400
167759	2018-May-10	2018-Jul-17	68	SE	41	M	3.5	Adult	116	28.1	383.7	814	216
168375	2018-May-10	2018-Jun-30	51	SE	73	M	3.2	Adult	168	21.5	386.4	286	80
174991	2018-May-10	2018-Jun-27	48	SE	53	F	4.0	Adult	18	51.5	192.9	1,446	64

Summary of tagging and tracking metrics for reef manta rays. Track period represents the number of days between the start date and end date (from tag deployment to last detection before tag shedding). Mons indicates the monsoon period during which the tag was deployed (NW Monsoon: November – April; SE Monsoon: May – October). % Dec shows the percentage of detection days within the track period. Sex, Disc Width (DW, m), and Maturity describe the physical and biological characteristics of each reef manta. # Positions gives the number of position estimates from Fastloc GPS and Argos locations. Max. Disp. (km) is the maximum recorded dispersal distance, Track Length is the total distance travelled between all position estimates, MCP (km²) is the area of the minimum convex polygon, and Max Depth is the maximum depth recorded by the tag.

**Fig 3:**
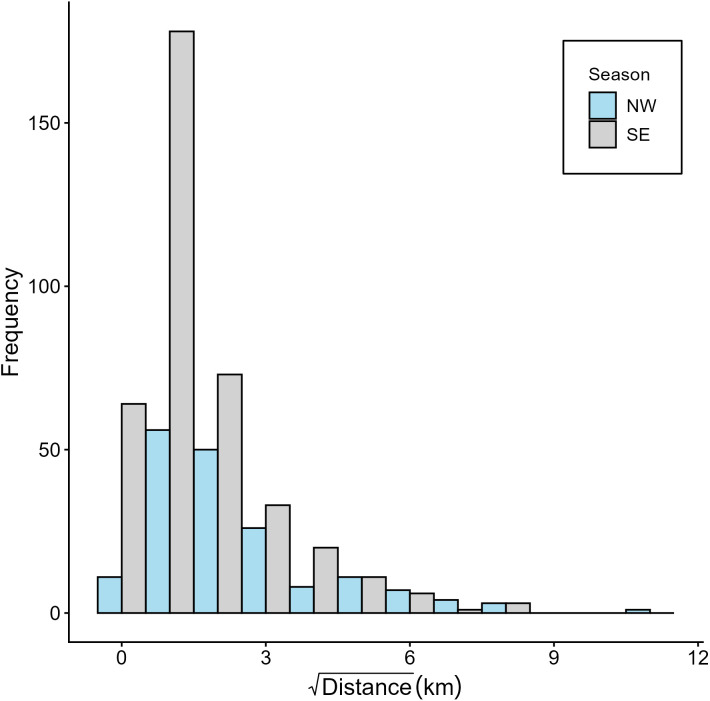
Movement patterns of tagged reef manta rays. Frequency of distances moved between relocations for the ten tagged reef manta rays in Milne Bay Province, Papua New Guinea during the NW and SE Monsoons. Distance is square-root transformed to reduce skew and improve visualization of the distribution across a wide range of movement values.

The mean displacement of position estimates of individuals from their respective tagging locations was 11.3 ± 14.7 km, with 75% of relocations occurring within ten kilometers and an overall maximum recorded displacement of 86.9 km (Table 1). The maximum track length was 862 km and occurred over a period of 146 days. The mean track length of all tags was 341.8 km (range 45.1–862 km) and overall, manta rays traveled an average of 4.8 ± 2.9 km per day at mean speeds of 0.66 ± 0.3 km/hr. When pooling data from all individuals, the distances moved between subsequent positions for each tag were higher during the NW Monsoon than SE Monsoon, with a mean of 9.32 km and 4.55 km, respectively (median values of 3.4 km and 1.5 km; Fig 3); however, this relationship was not statistically significantly different when accounting for individual variation (S2 Table).

**Fig 4:**
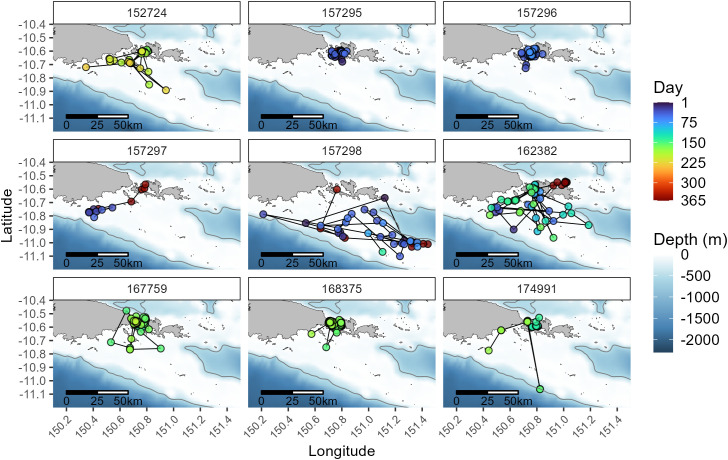
Track series of SPLASH-tagged reef manta rays. Fastloc GPS and Argos position estimates are shown for all individuals (excluding the 4-day track of 158697). Time series represents days from 1–365, corresponding to a Gregorian calendar year beginning in January and ending in December. The light grey line represents the 500 m isobath [38,39]. National boundaries were sourced from the Papua New Guinea National Statistics Office [34].

Collectively, individuals utilized habitats within the main islands of Sideia and Sariba across all months where tags reported position data and recorded surface temperatures between 25.0 and 30.8 °C. Tagged animals were detected along the main island chain as far west as Dagadaga Bonarua Island and as far east as the larger Basilaki Island (Moresby Island) on the corner of Kitai Katoa Island (Glenton Island) (Fig 1). The tagged cohort’s range further extended south and to the southeast towards the Papuan Plateau and was restricted horizontally within the 500 m isobath (Fig 4). Two individuals tagged in the NW Monsoon (IDs 157298 and 162382) had the longest deployment tracks and moved the furthest distances of all tracked animals from the initial deployment location (Table 1, Fig 4). ID 157298 was detected the furthest west at 150.21° E and east at 151.44° E, representing a Euclidian distance of 136.5 km. The high number of quality positions transmitted by ID’s 157298, 162382, 157274, and 157297 from the Papuan Plateau confirms site use of reef manta rays to this shelf habitat outside the geographical boundaries of the main Samarai Islands complex. The minimum convex polygons varied between 69–3,742 km^2^ for each individual and the overall 100% MCP for the tagged cohort was 5,274 km^2^.

**Fig 5:**
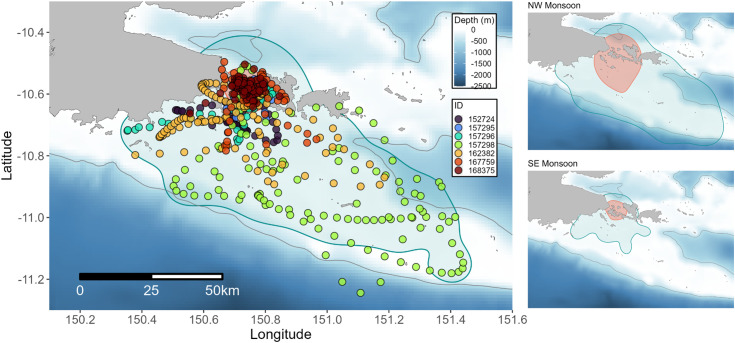
State-space modeled tracks and activity spaces of tagged reef manta rays. State-space modeled tracks of seven tagged reef manta rays fit using a random correlated walk and 12-hr time step. Kernel utilization distributions indicate the 95% (cyan) home range and 50% (red) core activity space using the interpolated track locations. The thin grey line represents the 500 m isobath [38,39]. National boundaries were sourced from the Papua New Guinea National Statistics Office [34].

**Fig 6:**
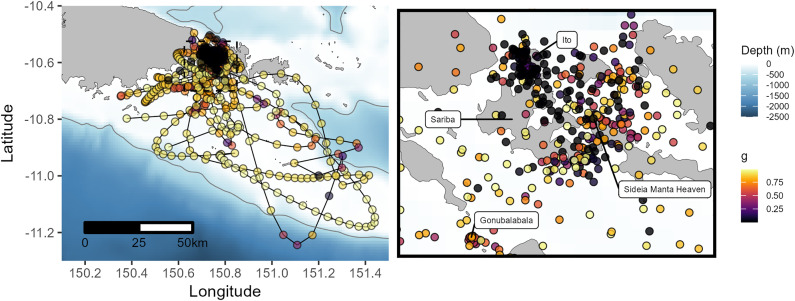
Movement persistence of reef manta rays based on state-space modeling. Resulting state-space modeled points of reef manta rays fitted with a move persistence index, *g*, from 0–1 where numbers closer to 1 indicate more transitory behavior. Higher numbers of *g* represent more transitory behavior while lower numbers represent more restricted movements. The right panel provides a close-up of the area outlined by the dashed black box in the left panel. The thin grey line on the left panel represents the 500 m isobath [38,39]. National boundaries were sourced from the Papua New Guinea National Statistics Office [34].

The state-space modeled tracks consisted of seven individuals and all movement models converged with no biologically implausible paths observed (S3 Table). The kernel utilization distribution incorporating points from all individuals estimated a 95% home range of 4,817.9 km^2^ and a core activity space (50% KUD) of 340.3 km^2^. This home range (95% KUD) of the collective cohort of tagged reef manta rays extended from Sideia Island in the north to the southcentral reefs and lagoons of the Milne Bay Province (Fig 5). The core activity space (50% KUD) included Ito Island and both tagging locations: Gonubalabala and Manta Sideia Heaven (Fig 5). During the NW monsoon, home range and core activity space expanded, encompassing the smaller areas observed during the SE monsoon without a significant directional shift (Fig 5). Overall core activity space overlapped with the majority of residency behavior states estimated by the move persistence model while transitory states were more prevalent along the Papuan Plateau (Fig 6).

**Fig 7:**
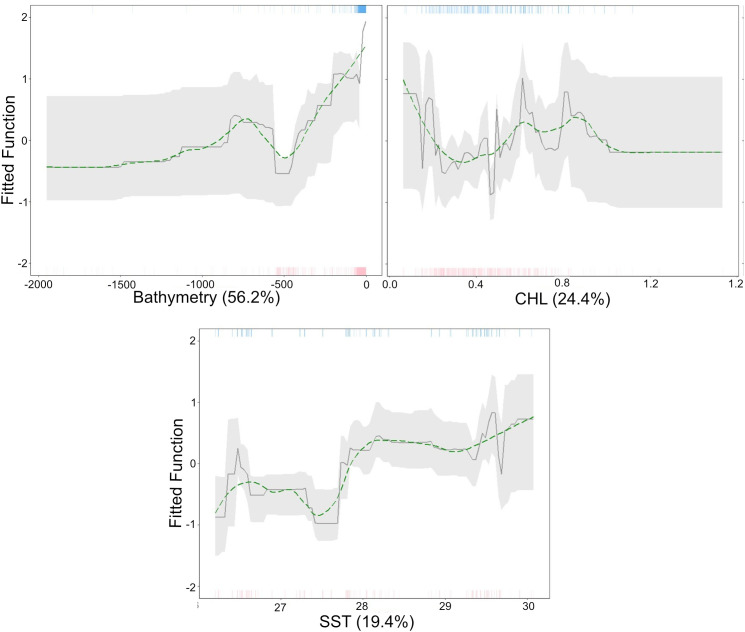
Environmental associations of reef manta rays from boosted regression tree modeling. Fitted functions of a boosted regression tree model describing associations between reef manta rays and three environmental co-variates: Bathymetry **(m)**, chlorophyll-a (CHL; mg/m^3^) and sea surface temperature (SST; °C). The green line shows smoothed partial dependency. Rugs display the distribution of the data for presence (top, blue), and absence (red, bottom).

Model performance evaluation for the BRT had outstanding predictive performance for the training (_T_AUC = 0.94) and cross-validated (_CV_AUC = 0.92) data, with minimal evidence of overfitting (ΔAUC = 0.08). The estimated *D*^2^ suggests that 47% of the deviance was explained (S3 Table). Relative contributions to manta ray presence from most to least influential co-variates included bathymetry (56.2%), chlorophyll-a concentration (24.4%), and SST (19.4%). Manta rays were more likely to be present at bathymetric depths of <250m with elevated chlorophyll and temperature values of approximately 0.6 mg/cm^3^ and 29.5 °C, respectively (Fig 7).

### Vertical occupancy

**Fig 8:**
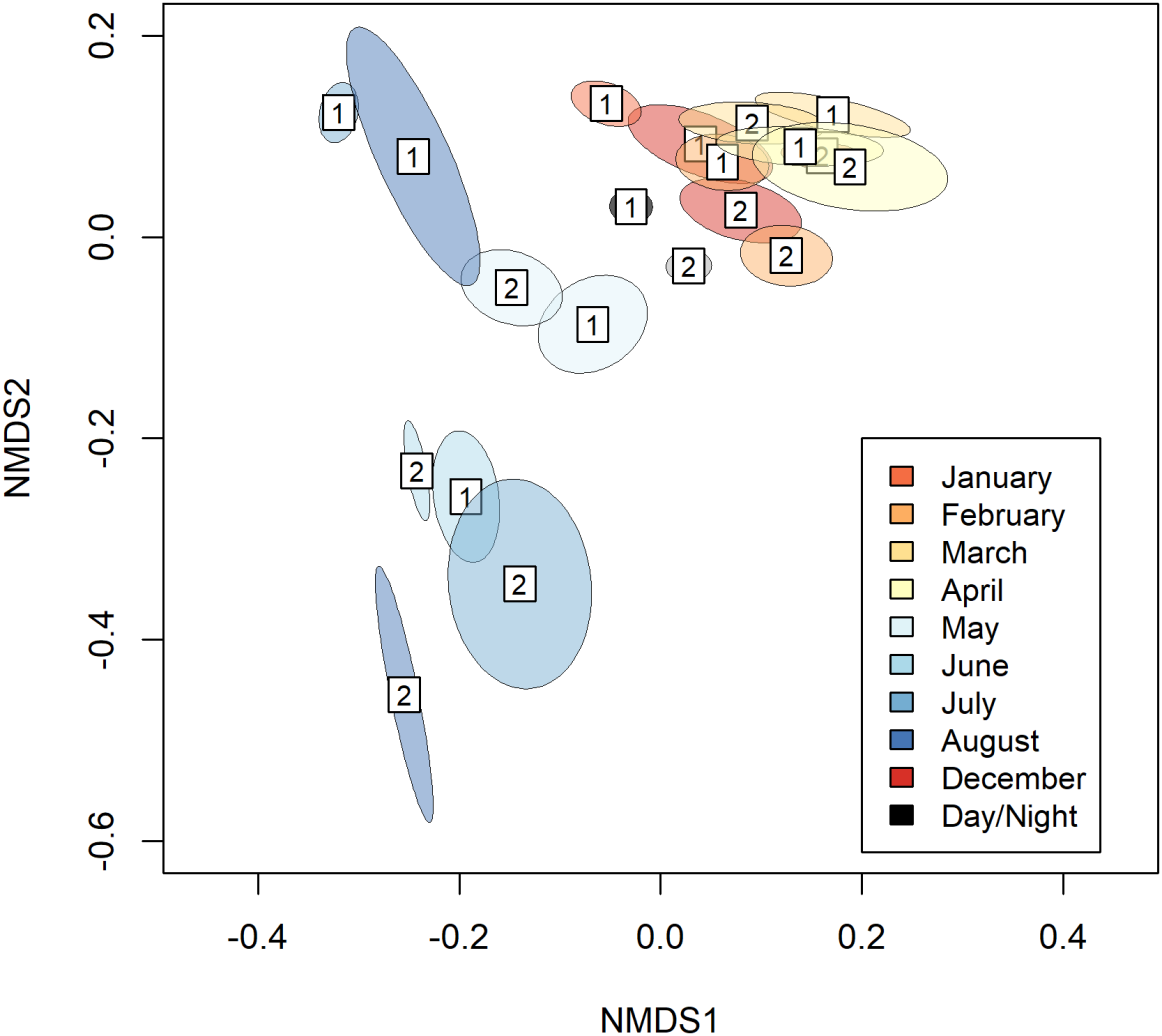
Multidimensional scaling of depth use by tagged reef manta rays. Non-parametric multidimensional scaling of ordinated 12-hr summarized depth histogram data obtained from SPLASH tagged reef manta rays in Milne Bay Province, Papua New Guinea. Labels 1 and 2 refer to the day and night time periods, respectively, while colors correspond with the months of tracking data.

**Fig 9:**
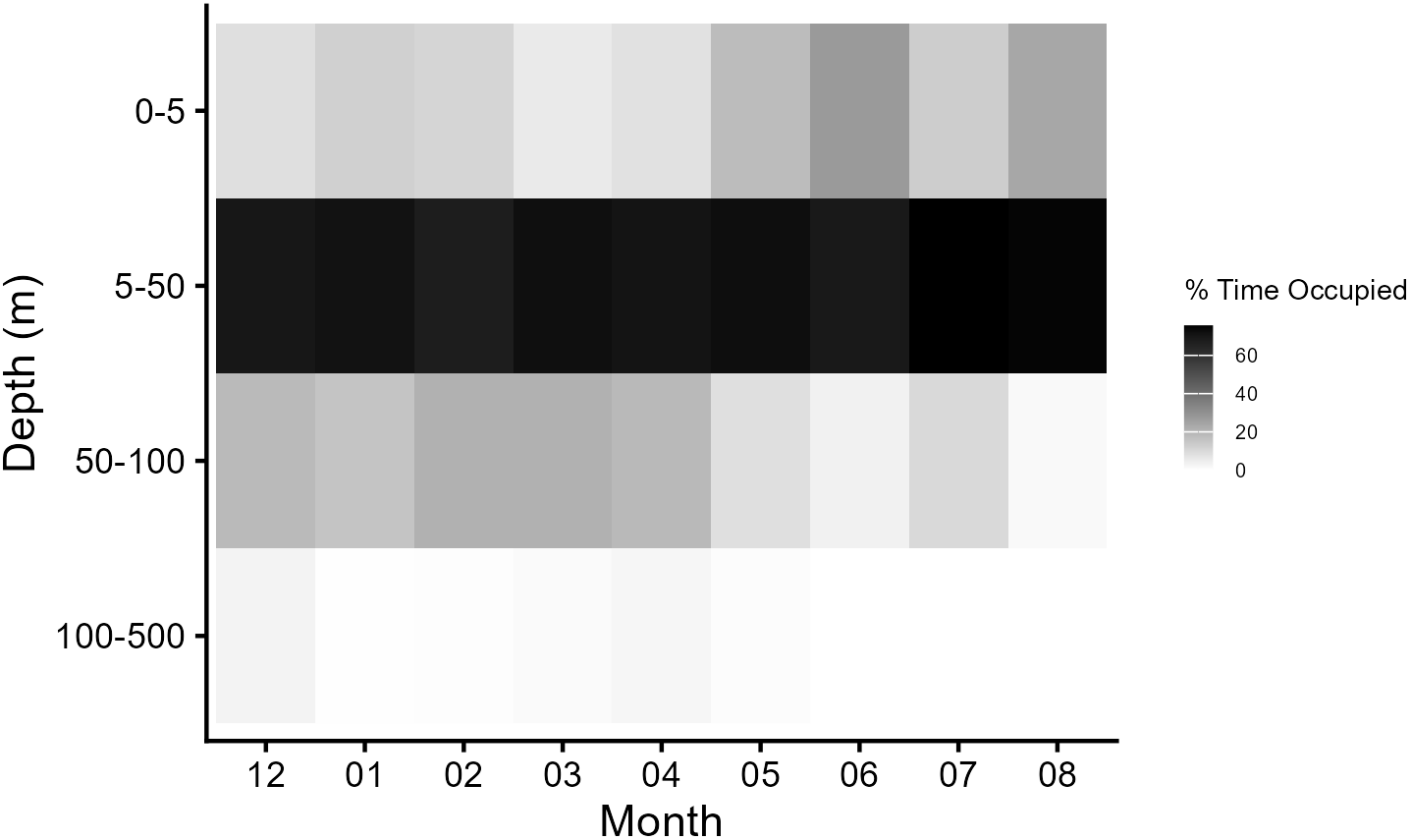
Depth use of tagged reef manta rays across monsoon seasons. Pooled histogram data for mean time spent within depth bins for each 12-hr period transmitted by seven tagged reef manta rays. Each individual was treated as a single replicate. The NW Monsoon months include dives from December – April and the SE Monsoon months included dives recorded in May – August.

Collectively, tagged manta rays spent 84.8 ± 11.3% and 93.1 ± 10.5% of their time in the upper 50 m of the water column during the NW and SE Monsoons, respectively. Time spent in each bin was significantly affected by month and the binary variable of day and night (F = 7.7436, p = 0.001). After depth data ordination, distinct clustering was apparent in the NMDS plot with the SE Monsoon months of May – August separating from the tightly clustered NW Monsoon months of December, January, February, March, and April (Fig 8). The nonoverlap in the clusters between monsoon months is highlighted in the heat map where reef manta rays on average spent higher amounts of time in the 50–100 m bin during the NW Monsoon and higher amounts of time within the collated 0–5 m bin during the SE Monsoon (Fig 9). Specifically, individuals tracked during the SE Monsoon spent approximately an average of 23.9 ± 11.5% of their time within 5 m of the surface as opposed to individuals tracked in the NW Monsoon that spent an average of 16.2 ± 14.6% of time there (Fig 9). Time spent between 50–100 m averaged 6.28 ± 9.08% and 14.5 ± 10.8% for the SE and NW Monsoons, respectively. However, GLMM models indicated that monsoon was only an important predictor for time spent above 5 m (β = 0.68 SE = 0.18, p = 0.00018; S4 Table) and not for time spent between 50–100 m (S5 Table).

**Fig 10:**
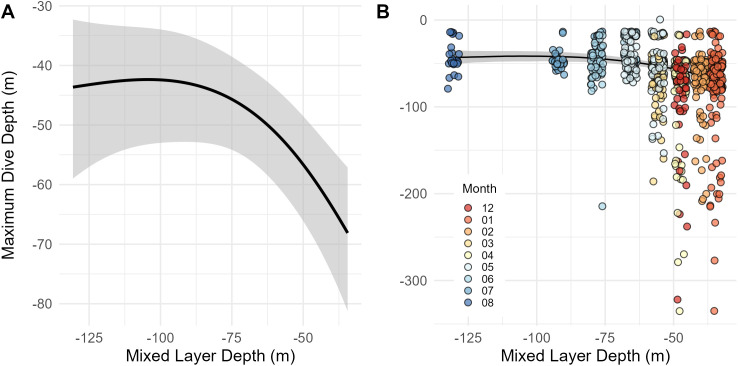
Relationship between maximum dive depth of reef manta rays and mixed layer depth. The black line shows the modeled relationship between maximum dive depth and mixed layer depth with (A) 95% confidence intervals and (B) bootstrapped 95% confidence intervals shaded in grey. **(B)** This relationship is plotted against raw values, which were jittered to improve visualization.

A total of 775 maximum dive depths were recorded and successfully transmitted across ten tagged individuals. Transmitted histograms indicated two individuals (157298 and 162382) reached a depth greater than 400 m during the month of December, but the maximum depth value for these points was not transmitted to the satellite. Only four individuals reached mesopelagic depths greater than 200 m and only 2.6% of all recorded dives reached these depths (n = 19), of which 18 occurred during the NW Monsoon. Deep dives reaching depths greater than 150 m were recorded 44 times across four individuals, with 42 of these dives occurring during the NW Monsoon months. Mean depth reached during all recorded dives was 63.2 ± 42.2 m (n = 775) with a mean nightly depth of 64.5 ± 42.4 m and mean daytime depth of 61.9 ± 41.9 m. When accounting for individual variation, the mean depth of these dives was 53.4 ± 14.0 m, with maximum nighttime depths (56.9 ± 15.0 m) greater than daytime depths (49.4 ± 15.8 m). The mean maximum dive depth during the NW Monsoon was greater than depths reached during the SE Monsoon, with an average of 63.0 ± 17.3 m and 48.4 ± 15.2 m across all individuals, respectively. The selected GAMM demonstrated a significant relationship between maximum dive depth and the mixed layer depth in the pooled data set, with deeper dives occurring at shallower mixed layer depths (edf = 2.2, F = 8.78, p < 0.001; S6 Table, Fig 10).

Of the eleven tagged reef manta rays, photo-IDs were obtained for ten. The only tagged individual without a photo-ID also failed to transmit any data (162381). Manta rays 152724, 157295, and 157296 were all resighted after approximately 18–24 months post-tagging at their tagging locations as observed by the authors in subsequent fieldwork expeditions.

## Discussion

### Movement and residency

The Samarai Islands and associated Papuan Plateau constitute critical habitat for reef manta rays, with tagged animals showing evidence of nearly year-round presence across two distinctive monsoonal periods and frequent use of areas around Ito Island, Sariba, Sideia, and Gonubalabala. Reef manta rays did not exhibit long-distance movements (i.e., > 100 km) away from their tagging site and 75% of relocations occurred within 10 km of the tagging site. Habitat use was confined to shallow bathymetry with no rays moving beyond the 500 m isobath. Additionally, no cross-over between reef manta rays from the Conflict Islands (located approximately 100 km away from the tagging locations) and those from the Samari Islands have been identified using limited photo-identification data (A. Murray, unpublished data). This site-attached behavior and limited long-distance movement resembles recent findings from SPLASH-tagged reef manta rays across multiple regions in Indonesia [57]. Reef manta ray movement patterns are highly context-dependent, with populations globally best described as partial migrators that display a wide spectrum of movement behaviors [58]. Although no long-distance movements were recorded from reef manta rays tagged in the Samarai Islands of Papua New Guinea, individuals in other populations are capable of straight-line movements of at least 500 km and even 1,150 km, as documented along the eastern and western coasts of Australia, within the Maldives Archipelago, and along the Mozambique-South Africa coastline [26,59–61]. Additionally, reef manta rays are also capable of long-distance dispersal, as evidenced by the sighting of a reef manta ray in Cocos Island, more than 6,000 km from the nearest known population [62]. It is important to note that many of these extreme long-distance movements have been observed through photo-identification, which can reveal connectivity over much longer timescales and broader spatial scales than typical telemetry deployments. However, some long-distance movements have also been captured using telemetry, with recorded movements up to 311 km from reef manta rays tagged in New Caledonia and ~700 km in Western Australia [59,63].

The wide variation in residency observed across reef manta ray populations, from isolated island archipelagos to coastlines with continuous reef habitat, highlights the complex and multi-faceted nature of their movement ecology [60,61,64–67]. This variation is probably driven by a combination of factors, including individual personality, regional oceanography, latitude, and the degree of shallow continuity between reefs [64,68]. For instance, the high residency observed in island systems may be driven by two primary mechanisms: increased localized productivity that is enhanced around oceanic islands in comparison to open ocean habitats (i.e., the Island Mass Effect), and the physical barrier of deep water that may limit wide-ranging dispersal [64]. The lack of such deep-water barriers along continuous coastlines could, in turn, provide more extensive habitats for long-distance movements, resulting in a comparatively higher proportion of long-distance migrators in these systems. Additionally, populations at higher latitudes, which are exposed to more extreme seasonal changes may migrate further to exploit seasonal resources, while tropical populations may make shorter, localized movements in response to less extreme seasonal shifts [68].

The Samarai Islands and surrounding region present a unique set of oceanographic conditions that could influence reef manta ray movement. While the area is best described as a continental shelf with shallow topography (the Papuan Plateau) that provides coastal connectivity to the rest of Papua New Guinea, it is also a low-latitude system with lower exposure to the extreme seasonal changes that could be driving extensive movements for populations at higher latitudes. Some long-distance movements might be expected due to the potential for dispersal along the continental shelf, none were observed in the current study. Instead, tagged reef manta rays showed consistent use of the Samarai Islands and Papuan Plateau during the collective eight month-tracking period. While seasonal changes in acoustic telemetry detections have been observed in several Indian Ocean populations [6,65,69–71], concrete evidence of distinct horizontal habitat switching where animals physically displace their core use areas is demonstrated well in the Maldives. Reef manta rays exhibit clear biannual habitat switching in the Maldives, where they move to the leeward sides of islands and reefs where primary production is influenced by the prevailing winds [6,70]. In contrast to this example of horizontal displacement, our study found that while core activity space and home range expanded during the NW Monsoon compared to the SE Monsoon, these areas did not exhibit a significant horizontal shift, but rather represented an expansion of existing ranges.

### Diving behavior and oceanographic linkages

Concurrently collected depth data from the current study suggested tagged rays may have altered their vertical diving behavior between monsoons, which could indicate that they strategically locate the best foraging opportunities in the vertical dimension, negating the need to undergo longer horizontal migrations to find such resources. Specifically, maximum diving depths were positively associated with shallower mixed layer depths and rays exhibited higher occupancy of surface habitats during the SE Monsoon as opposed to the NW Monsoon. The Samarai Islands sit along an upwelling zone on the northern edge of the Coral Sea, and reef manta rays have access to shallow and deep-water habitats within 50 km of the tagging site where ample opportunities may exist for foraging throughout the year. This capacity for behavioral plasticity in vertical distribution has also been documented in oceanic manta rays (*Mobula birostris*) in the Revillagigedo Archipelago [72] and has been suggested to occur in reef manta rays [59,71]. Similar vertical adjustments have also been observed in other marine megaplanktivores that maintain residency in a specific area. For instance, an apparent seasonal aggregation of whale sharks (*Rhincodon typus*) in Mafia Island, Tanzania was found to be present throughout the year, with whale sharks switching between deeper forereef habitats and shallow surface waters on a biannual basis [73].

The observed vertical distribution shift aligns with seasonal oceanographic changes that may be driving surface productivity, such as sea surface temperature and wind strength. Our modeled mixed layer depth data supports this; the NW Monsoon coincides with a shallower MLD and increased stratification. Heightened stratification reduces vertical mixing and suppresses the movement of nutrients from bottom layers to oligotrophic surface layers, thereby limiting phytoplankton production [74,75]. We found that the shallowing of the MLD during the NW Monsoon coincided with a drop in satellite sensed chlorophyll-a concentration levels in the Sideia-Sariba complex and along the shelf, mirroring observations in the South China Sea where deeper mixed layers correlated with higher surface chlorophyll-a and phytoplankton biomass [76]. The MLD in this region of Papua New Guinea is primarily influenced by changes in the prevailing southeasterly trade winds, which are significantly stronger during the SE Monsoon than the NW Monsoon [35].

The southeasterly trade wind can create optimal surface foraging habitat for reef manta rays in two ways. Firstly, prevailing southeasterly winds can push surface water towards the northwest, driving the upwelling of cooler, nutrient-rich water from the Coral Sea via Ekman transport, deepening the MLD through mechanical wind shearing, and ultimately increasing primary productivity in the region of Milne Bay. Secondly, vertically migrating zooplankton may be pushed by these winds from the southeast direction onto the shallow shelf and reefs around the islands of Sideia and Sariba and become trapped by the shallow bathymetry. Alternatively, during the NW Monsoon the prevailing wind direction becomes more variable, with winds coming more from the West and North [35]. These winds are also generally less strong, which is associated with reduced upwelling. Reef manta rays may be forced to seek out mesopelagic prey in the deep scattering layer more often to compensate for reduced zooplankton patches in surface ocean layers, which might explain the movements of tagged individuals 162382, 157297, and 157298 towards the shelf during the NW Monsoon and the increase in the number of deeper dives performed.

Despite this tendency for reef manta rays to reach deeper depths during the NW Monsoon, there appears to be individual-level variability, possibly due to intra-specific competition for limited resources or the presence of localized refugia. For example, individuals 157295 and 157296 remained within the northern island complex in the NW Monsoon, indicating this area could be an important refuge for the species during months when region-wide chlorophyll levels are low and the MLD is shallow. Additionally, while there are clear distinctions in oceanographic conditions between the NW and SE Monsoons in the Samarai Islands, this area has less surface warming compared to the wider region during the NW Monsoon, which may contribute to the observed residency and low dispersal.

### Important habitat and conservation management implications

Channels served as important hotspots for reef manta ray activity within the study area, with frequent positions recorded around Ito, between Sariba and Galahi, and between Sariba and Sideia. These narrow waterways often concentrate zooplankton, attracting manta rays for feeding opportunities, particularly during ebb tides [77,78]. Residency indices revealed a strong association with the island groups, while more transitory behavior was observed towards the Papuan Plateau. Notably, the 50% core KUD encompassed the Sideia-Sariba channel and greater island complex during both the NW and SE monsoon periods, aligning closely with areas where direct observations during fieldwork showed manta rays feeding and engaging in cleaning behavior on the reef.The high density of satellite positions at other sites (Ito and Sariba-Galahi) indicates additional important habitats, but in-water behavior at these locations has not yet been validated. Reef manta rays are especially vulnerable to anthropogenic threats, especially in channels where these animals either aggregate to forage on trapped zooplankton patches, clean on coral bommies, or move through narrow habitats on their passage to other areas [31,71,79]. Community-based monitoring could help identify local impacts and locally agreed-upon measures could mitigate such risks.

The lack of recorded long-distance movements by this population and high site affinity with this region suggests that conservation measures implemented through local management alone are likely to be successful. Papua New Guinea is a signatory to both the Convention on International Trade in Endangered Species and the Convention on the Conservation of Migratory Species, and the country currently does not have any national protections for mobulid rays. Collision with shipping vessels along highly trafficked trade routes is suspected to be a source of cryptic mortality for migratory marine species, such as whale sharks [80]. While a major N-S shipping route connecting Asia to Eastern Australian port cities passes through the general region at approximately 152.5 °E, no reef manta rays tagged in the Samarai Islands were detected this far east. Additionally, reef manta rays spent a majority of their time in subsurface waters between 5–50 m, which would reduce their overall risk of vessel collision. Bycatch of reef manta rays from the prawn trawl fishery continues to be a concern for reef manta rays in Papua New Guinea but these trawlers do not operate on the part of the Papuan Plateau described in this study nor do they operate in the vicinity of the Samarai Islands [24]. However, evidence of targeted manta ray hunting was documented at the Dumoulin Islands in May 2016 (Erdmann, pers. obs.), where four manta ray carcasses (heads only) were observed alongside finned shark carcasses, indicating that directed fishing has occurred and may continue to pose a threat to the population. Such activities appear to be localized and are not widely reported by local fishers or dive guides, suggesting that targeted hunting pressure remains relatively low across the broader study region. While this represents a potential ongoing threat that requires monitoring, the apparently limited scale of such activities indicates that local conservation measures could still be effective for population management.

Overall, lethal impacts on the population studied here appear to be isolated or infrequent [81], presenting an opportunity to proactively develop manta ray-based ecotourism in the region. To ensure sustainable and non-disruptive interactions between tourists and reef manta rays, the implementation of scientifically informed, adaptive management strategies is essential. A pilot ecotourism program could be developed based on successful frameworks establish at other manta dive sites, incorporating measures such as a maximum number of divers per encounter, minimum approach distances, time limits for in-water interactions, and seasonal closures during sensitive periods [17]. Globally, tourism has the potential to negatively impact reef manta ray behavior, for example, in Baa Atoll, Maldives [17] and Western Australia [82], highlighting the importance of early evidence-based planning. By carefully managing visitation, new manta ray ecotourism sites, such as those in Papua New Guinea, may help alleviate pressure on overexploited areas while promoting conservation awareness and local economic benefits, without compromising the fitness or behavior of the target population.

### Limitations and future research

Our interpretation of vertical distribution should be evaluated with caution as it is based on a small sample size (n = 10) and low-resolution vertical distribution data sourced from transmitted histogram and maximum diving depth data. Nonetheless, barring the opportunistic retrieval of archived data, our data offers preliminary insights into the vertical behavioral plasticity of this population. With the limitations of a short sampling duration and sample size, it is possible that we simply missed tagging an animal with a higher propensity to migrate further distances or missed observing more extensive horizontal shifts. Therefore, a more comprehensive understanding of this population’s residency dynamics would benefit from an acoustic tagging study that would enable a higher sampling size, longer rate of retention compared to towed tags, and a broader representation of demographic classes for analyses. Additionally, methods that lack the temporal sampling constraints of telemetry, such as photo-ID and genetics approaches, can be used to ascertain connectivity with the nearby Conflict Islands group and the broad-scale home range of the Samarai Islands’ population. Furthermore, future research should aim to collect muscle and skin samples from manta rays throughout the year to test the hypothesis of diet switching between demersal, epipelagic, and mesopelagic zooplankton prey, which could provide further insight into foraging strategies in response to environmental variability.

## Supporting information

S1 Fig Sea surface trends in the study region. Monthly averaged sea surface temperature (SST °C) of the region during the study period from 2016–2018 with the study area located within the dashed black box. National boundaries were sourced from the Papua New Guinea National Statistics Office [34].
(TIFF)

S2 Fig Chlorophyll-a dynamics around the Samari Islands. Monthly mean composites of chlorophyll-a concentration (mg/m3) across the study period of the Samarai Islands, 2016 – 2018. National boundaries were sourced from the Papua New Guinea National Statistics Office [34].
(TIFF)

S3 Fig Mixed layer depth variability in the study area. Mean monthly mixed layer depth across the study period, 2016 –2018, determined to be the depth at which the temperature decreases by one degree °C compared to the temperature at 10m depth.
(TIFF)

S4 Fig Temporal coverage of tagged reef manta rays. The temporal coverage of responding tags and their transmitted horizontal locations from tagged reef manta rays in the Samarai Islands of Milne Bay Province. The blue shaded area represents the SE Monsoon and unshaded areas correspond to the NW Monsoon.
(TIFF)

S1 Table Boosted regression tree model calibration results. Boosted regression tree model results for 36 tested models with differing tree complexity (tc), learning rates (lr), bag fraction (bf) and step size (ss) Model performance metrics are shown as TAUC = Training AUC; CVAUC = Cross Validation AUC; D2 = Deviance explained, with the best performing model highlighted in bold.
(DOCX)

S2 Table Model selection results for movement distances of reef manta rays. Selection table for generalized linear mixed models used to evaluate the relationship between the distance between relocated locations of SPLASH tagged reef manta rays (n = 10) and the monsoon period. The chosen model is bolded. Column names correspond to the following: df = degrees of freedom, AICc = Akaike information criterion corrected for sample size, ΔAICc = the difference in the AICc, wAICc = AICc weight, R2 Cond = the proportion of variance explained by fixed and random effects, and R2 Marg = the proportion of variance explained by fixed effects.
(DOCX)

S3 Table Summary of state-space model outputs for tagged individuals. Values include the number of locations used to fit the models (n.filtered), number of predicted positions (n.fit) and convergence status. Tracks were segmented if they contained gaps of more than one week without a recorded position and then removed if those segments contained less than 10 detections.
(DOCX)

S4 Table Model selection for time spent in 0–5 m depth bin. Selection table for generalized linear mixed models used to evaluate the relationship between the proportion of time spent in depth bins between 0–5 m and the monsoon period. The chosen model is bolded. Column names correspond to the following: df = degrees of freedom, AICc = Akaike information criterion corrected for sample size, ΔAICc = the difference in the AICc, wAICc = AICc weight, R2 Cond = the proportion of variance explained by fixed and random effects, and R2 Marg = the proportion of variance explained by fixed effects.
(DOCX)

S5 Table Model selection for time spent in 50–100 m depth bin. Selection table for generalized linear mixed models used to evaluate the relationship between the proportion of time spent in depth bins between 50–100 m and the monsoon period. The chosen model is bolded. Column names correspond to the following: df = degrees of freedom, AICc = Akaike information criterion corrected for sample size, ΔAICc = the difference in the AICc, wAICc = AICc weight, R2 Cond = the proportion of variance explained by fixed and random effects, and R2 Marg = the proportion of variance explained by fixed effects.
(DOCX)

S6 Table Model selection for relationship between diving depth and mixed layer depth. Selection table for generalized additive mixed models used to evaluate the relationship between the maximum diving depth of SPLASH tagged reef manta rays and the estimated monthly mixed layer depth of the region. The chosen model is bolded. Column names correspond to the following: df = degrees of freedom, AICc = Akaike information criterion corrected for sample size, ΔAICc = the difference in the AICc, wAICc = AICc weight, R2 = the proportion of variance explained by fixed and random effects.
(DOCX)

S1 Data Data. (XLSX)
