# Supplementary figures and images for: Movement, residency, and behavioral plasticity of reef manta rays in the Samarai Islands of Papua New Guinea
Source: PLoS One. 2026 May 28;21(5):e0344615. doi: 10.1371/journal.pone.0344615 (PMC13218459; doi:10.1371/journal.pone.0344615)

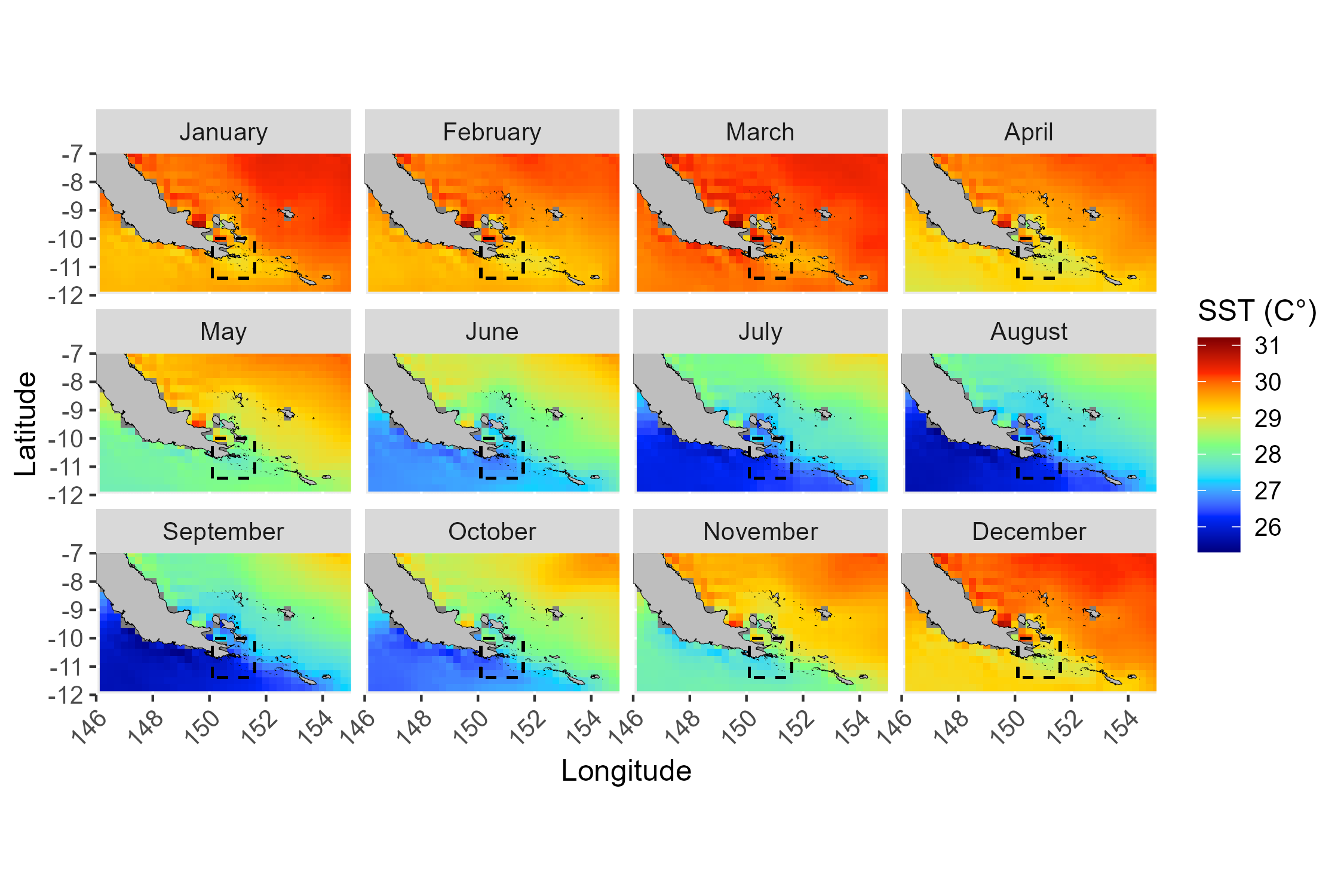

Supplement: S1 Fig — Monthly averaged sea surface temperature (SST °C) of the region during the study period from 2016–2018 with the study area located within the dashed black box. National boundaries were sourced from the Papua New Guinea National Statistics Office [34]. (TIFF) [file pone.0344615.s001.tiff]

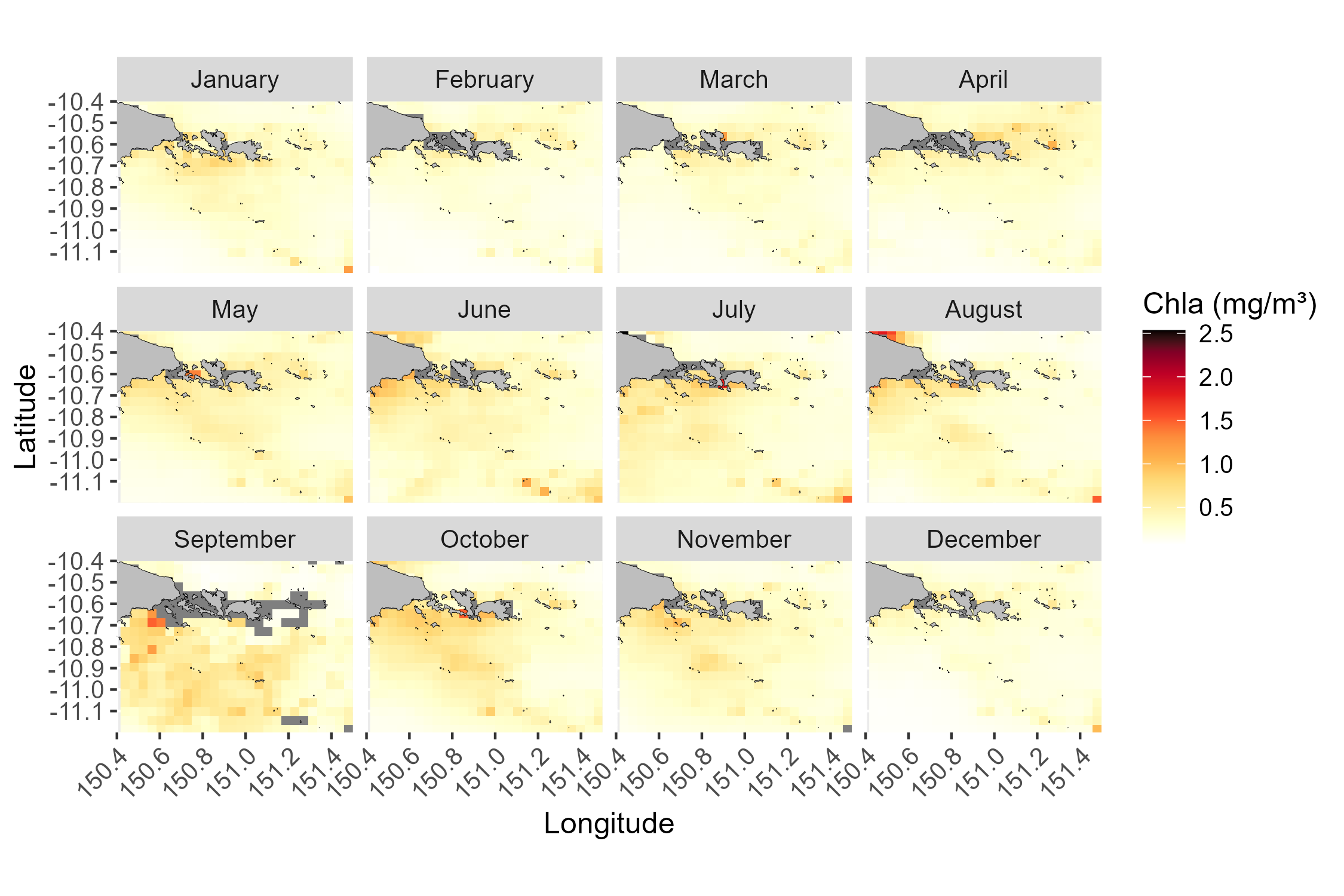

Supplement: S2 Fig — Monthly mean composites of chlorophyll-a concentration (mg/m3) across the study period of the Samarai Islands, 2016 – 2018. National boundaries were sourced from the Papua New Guinea National Statistics Office [34]. (TIFF) [file pone.0344615.s002.tiff]

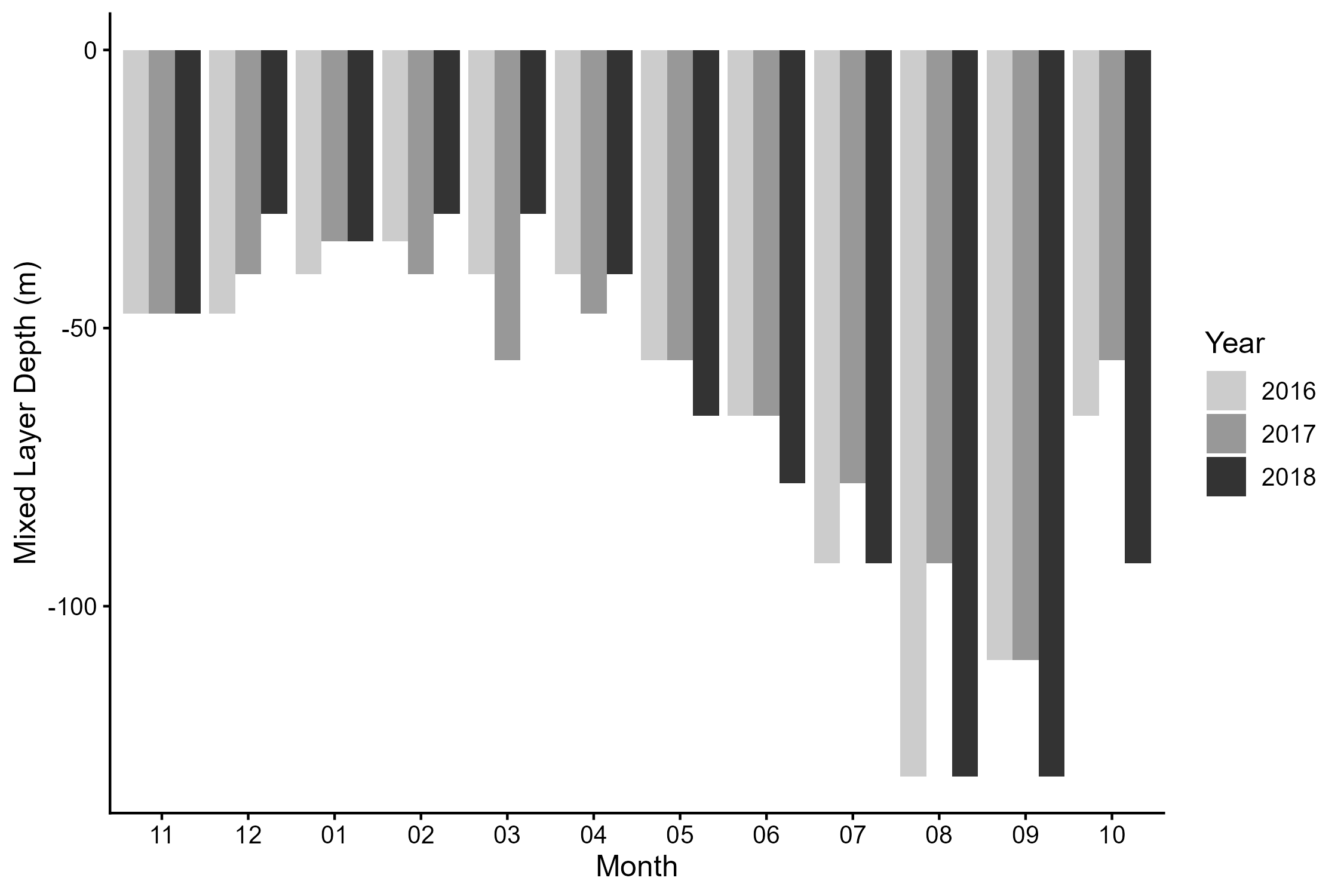

Supplement: S3 Fig — Mean monthly mixed layer depth across the study period, 2016 –2018, determined to be the depth at which the temperature decreases by one degree °C compared to the temperature at 10m depth. (TIFF) [file pone.0344615.s003.tiff]

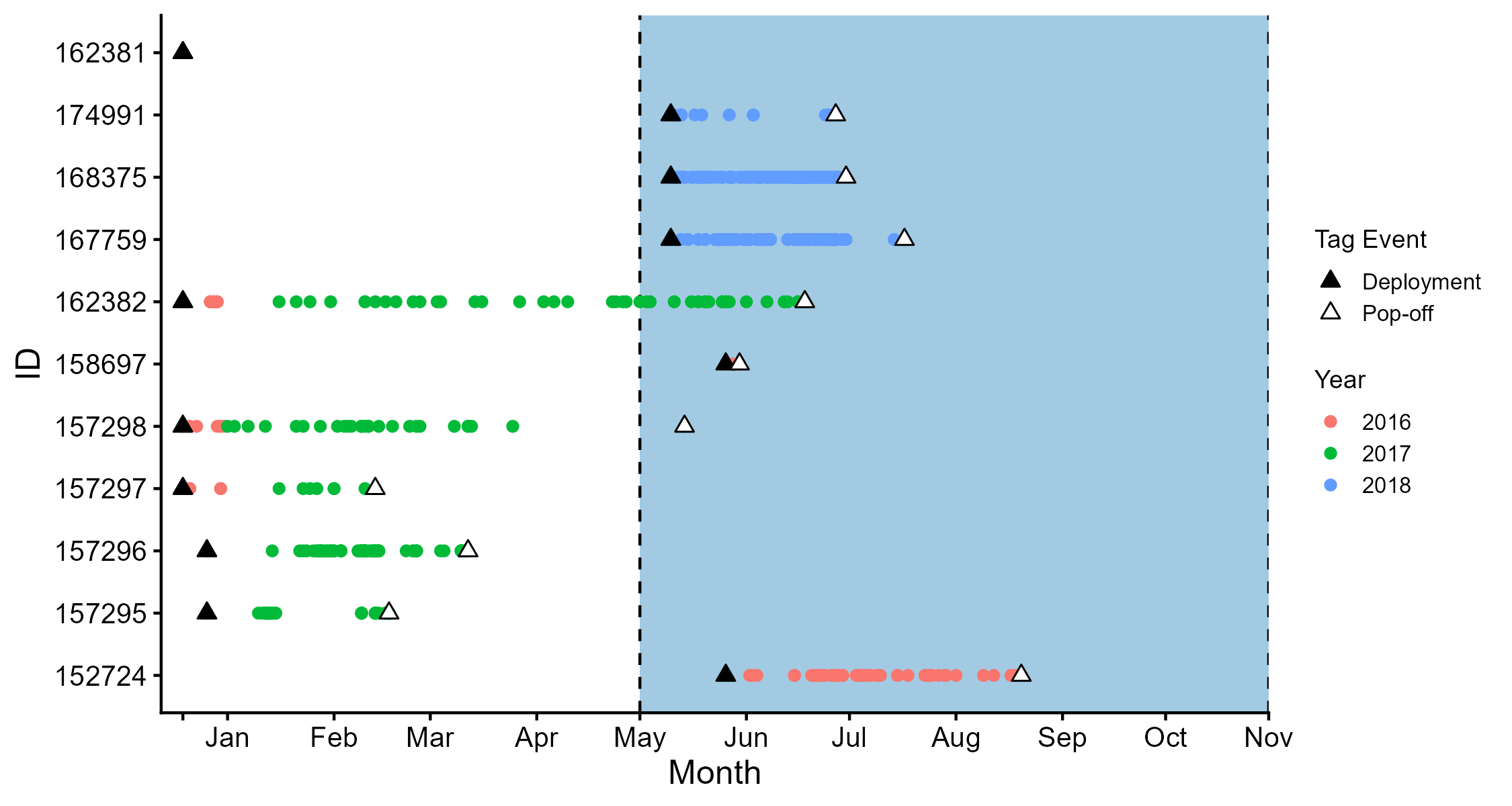

Supplement: S4 Fig — The temporal coverage of responding tags and their transmitted horizontal locations from tagged reef manta rays in the Samarai Islands of Milne Bay Province. The blue shaded area represents the SE Monsoon and unshaded areas correspond to the NW Monsoon. (TIFF) [file pone.0344615.s004.tiff]
