# Supplementary material for: Movement, residency, and behavioral plasticity of reef manta rays in the Samarai Islands of Papua New Guinea
Source: PLoS One. 2026 May 28;21(5):e0344615. doi: 10.1371/journal.pone.0344615 (PMC13218459; doi:10.1371/journal.pone.0344615)
Supplement: S1 Table — Boosted regression tree model results for 36 tested models with differing tree complexity (tc), learning rates (lr), bag fraction (bf) and step size (ss) Model performance metrics are shown as TAUC = Training AUC; CVAUC = Cross Validation AUC; D2 = Deviance explained, with the best performing model highlighted in bold. (DOCX) [file pone.0344615.s005.docx]

**Table S1.** **Boosted regression tree model calibration results.** Boosted regression tree model results for 36 tested models with differing tree complexity (tc), learning rates (lr), bag fraction (bf) and step size (ss) Model performance metrics are shown as TAUC = Training AUC; CVAUC = Cross Validation AUC; D2 = Deviance explained, with the best performing model highlighted in bold.

| **Model** | **tc** | **lr** | **bf** | **ss** | **Total Deviance** | **Residual Deviance** | **Correlation** | **cvDeviance** | **cvCorrelation** | **_T_AUC** | **_CV_AUC** | **ΔAUC** | ***D^2^*** |
| --- | --- | --- | --- | --- | --- | --- | --- | --- | --- | --- | --- | --- | --- |
| 1 | 1 | 0.01 | 0.5 | 25 | 1.39 | 0.93 | 0.61 | 1.00 | 0.57 | 0.85 | 0.82 | 0.03 | 32.9 |
| 2 | 1 | 0.01 | 0.5 | 50 | 1.39 | 0.92 | 0.62 | 1.00 | 0.57 | 0.86 | 0.82 | 0.03 | 33.4 |
| 3 | 1 | 0.01 | 0.7 | 25 | 1.39 | 0.94 | 0.61 | 1.00 | 0.56 | 0.85 | 0.82 | 0.03 | 32.0 |
| 4 | 1 | 0.01 | 0.7 | 50 | 1.39 | 0.94 | 0.61 | 1.01 | 0.56 | 0.85 | 0.82 | 0.03 | 32.2 |
| 5 | 1 | 0.005 | 0.5 | 25 | 1.39 | 0.96 | 0.60 | 1.01 | 0.56 | 0.84 | 0.82 | 0.02 | 31.1 |
| 6 | 1 | 0.005 | 0.5 | 50 | 1.39 | 0.93 | 0.61 | 1.00 | 0.57 | 0.85 | 0.82 | 0.03 | 32.7 |
| 7 | 1 | 0.005 | 0.7 | 25 | 1.39 | 0.96 | 0.60 | 1.01 | 0.56 | 0.84 | 0.82 | 0.02 | 31.0 |
| 8 | 1 | 0.005 | 0.7 | 50 | 1.39 | 0.93 | 0.61 | 0.99 | 0.57 | 0.85 | 0.82 | 0.03 | 32.7 |
| 9 | 1 | 0.001 | 0.5 | 25 | 1.39 | 0.99 | 0.58 | 1.02 | 0.56 | 0.83 | 0.82 | 0.01 | 28.6 |
| 10 | 1 | 0.001 | 0.5 | 50 | 1.39 | 0.97 | 0.59 | 1.01 | 0.56 | 0.84 | 0.82 | 0.02 | 29.8 |
| 11 | 1 | 0.001 | 0.7 | 25 | 1.39 | 0.99 | 0.58 | 1.02 | 0.56 | 0.83 | 0.82 | 0.01 | 28.8 |
| 12 | 1 | 0.001 | 0.7 | 50 | 1.39 | 0.97 | 0.59 | 1.01 | 0.56 | 0.84 | 0.82 | 0.01 | 29.8 |
| 13 | 2 | 0.01 | 0.5 | 25 | 1.39 | 0.84 | 0.68 | 0.97 | 0.59 | 0.89 | 0.83 | 0.06 | 39.7 |
| 14 | 2 | 0.01 | 0.5 | 50 | 1.39 | 0.74 | 0.73 | 0.96 | 0.60 | 0.92 | 0.84 | 0.08 | 46.6 |
| 15 | 2 | 0.01 | 0.7 | 25 | 1.39 | 0.84 | 0.67 | 0.97 | 0.59 | 0.89 | 0.83 | 0.06 | 39.3 |
| 16 | 2 | 0.01 | 0.7 | 50 | 1.39 | 0.78 | 0.71 | 0.97 | 0.59 | 0.91 | 0.83 | 0.08 | 43.6 |
| 17 | 2 | 0.005 | 0.5 | 25 | 1.39 | 0.86 | 0.66 | 0.97 | 0.58 | 0.88 | 0.83 | 0.05 | 37.9 |
| 18 | 2 | 0.005 | 0.5 | 50 | 1.39 | 0.80 | 0.70 | 0.96 | 0.59 | 0.90 | 0.83 | 0.07 | 42.1 |
| 19 | 2 | 0.005 | 0.7 | 25 | 1.39 | 0.85 | 0.67 | 0.98 | 0.58 | 0.89 | 0.83 | 0.06 | 38.5 |
| 20 | 2 | 0.005 | 0.7 | 50 | 1.39 | 0.78 | 0.71 | 0.96 | 0.59 | 0.91 | 0.84 | 0.07 | 43.7 |
| 21 | 2 | 0.001 | 0.5 | 25 | 1.39 | 0.93 | 0.62 | 0.99 | 0.58 | 0.86 | 0.83 | 0.03 | 33.0 |
| 22 | 2 | 0.001 | 0.5 | 50 | 1.39 | 0.90 | 0.64 | 0.98 | 0.58 | 0.87 | 0.83 | 0.04 | 35.2 |
| 23 | 2 | 0.001 | 0.7 | 25 | 1.39 | 0.94 | 0.62 | 1.00 | 0.57 | 0.85 | 0.82 | 0.03 | 32.4 |
| 24 | 2 | 0.001 | 0.7 | 50 | 1.39 | 0.90 | 0.64 | 0.98 | 0.58 | 0.87 | 0.83 | 0.04 | 35.1 |
| 25 | 3 | 0.01 | 0.5 | 25 | 1.39 | 0.77 | 0.72 | 0.95 | 0.60 | 0.91 | 0.84 | 0.07 | 44.4 |
| 26 | 3 | 0.01 | 0.5 | 50 | 1.39 | 0.74 | 0.74 | 0.96 | 0.59 | 0.92 | 0.84 | 0.09 | 46.8 |
| **27** | **3** | **0.01** | **0.7** | **25** | **1.39** | **0.74** | **0.74** | **0.94** | **0.61** | **0.92** | **0.85** | **0.08** | **46.7** |
| 28 | 3 | 0.01 | 0.7 | 50 | 1.39 | 0.75 | 0.73 | 0.96 | 0.59 | 0.92 | 0.84 | 0.08 | 45.7 |
| 29 | 3 | 0.005 | 0.5 | 25 | 1.39 | 0.80 | 0.70 | 0.96 | 0.59 | 0.91 | 0.84 | 0.07 | 42.5 |
| 30 | 3 | 0.005 | 0.5 | 50 | 1.39 | 0.78 | 0.71 | 0.96 | 0.60 | 0.91 | 0.84 | 0.07 | 43.6 |
| 31 | 3 | 0.005 | 0.7 | 25 | 1.39 | 0.81 | 0.70 | 0.96 | 0.59 | 0.90 | 0.84 | 0.07 | 41.6 |
| 32 | 3 | 0.005 | 0.7 | 50 | 1.39 | 0.74 | 0.74 | 0.95 | 0.59 | 0.93 | 0.84 | 0.09 | 47.0 |
| 33 | 3 | 0.001 | 0.5 | 25 | 1.39 | 0.90 | 0.64 | 0.98 | 0.58 | 0.87 | 0.83 | 0.04 | 35.0 |
| 34 | 3 | 0.001 | 0.5 | 50 | 1.39 | 0.86 | 0.66 | 0.98 | 0.58 | 0.88 | 0.83 | 0.05 | 37.9 |
| 35 | 3 | 0.001 | 0.7 | 25 | 1.39 | 0.89 | 0.65 | 0.98 | 0.58 | 0.87 | 0.83 | 0.04 | 35.5 |
| 36 | 3 | 0.001 | 0.7 | 50 | 1.39 | 0.84 | 0.68 | 0.97 | 0.59 | 0.89 | 0.83 | 0.06 | 39.3 |
