# Supplementary material for: Movement, residency, and behavioral plasticity of reef manta rays in the Samarai Islands of Papua New Guinea
Source: PLoS One. 2026 May 28;21(5):e0344615. doi: 10.1371/journal.pone.0344615 (PMC13218459; doi:10.1371/journal.pone.0344615)
Supplement: S3 Table — Values include the number of locations used to fit the models (n.filtered), number of predicted positions (n.fit) and convergence status. Tracks were segmented if they contained gaps of more than one week without a recorded position and then removed if those segments contained less than 10 detections. (DOCX) [file pone.0344615.s007.docx]

**Table S3. Summary of state-space model outputs for tagged individuals.** Values include the number of locations used to fit the models (n.filtered), number of predicted positions (n.fit) and convergence status. Tracks were segmented if they contained gaps of more than one week without a recorded position and then removed if those segments contained less than 10 detections.

| **ID** | **Model** | **Time** | **n.filtered** | **n.fit** | **Converged** | **AICc** |
| --- | --- | --- | --- | --- | --- | --- |
| 152724_3 | Crw | 12 | 45 | 95 | True | 463.2 |
| 157295_2 | Crw | 12 | 13 | 10 | True | 130.8 |
| 157295_3 | Crw | 12 | 15 | 18 | True | 138.6 |
| 157296_3 | Crw | 12 | 45 | 49 | True | 485 |
| 157296_4 | crw | 12 | 14 | 36 | True | 174.9 |
| 157298_1 | Crw | 12 | 10 | 50 | True | 197.5 |
| 157298_2 | Crw | 12 | 14 | 74 | True | 304.3 |
| 162382_8 | Crw | 12 | 25 | 114 | True | 405.8 |
| 167759_1 | Crw | 12 | 109 | 104 | True | 1092.8 |
| 168375_1 | Crw | 12 | 162 | 103 | True | 1452.9 |
