# Supplementary material for: Movement, residency, and behavioral plasticity of reef manta rays in the Samarai Islands of Papua New Guinea
Source: PLoS One. 2026 May 28;21(5):e0344615. doi: 10.1371/journal.pone.0344615 (PMC13218459; doi:10.1371/journal.pone.0344615)
Supplement: S4 Table — Selection table for generalized linear mixed models used to evaluate the relationship between the proportion of time spent in depth bins between 0–5 m and the monsoon period. The chosen model is bolded. Column names correspond to the following: df = degrees of freedom, AICc = Akaike information criterion corrected for sample size, ΔAICc = the difference in the AICc, wAICc = AICc weight, R2 Cond = the proportion of variance explained by fixed and random effects, and R2 Marg = the proportion of variance explained by fixed effects. (DOCX) [file pone.0344615.s008.docx]

**Table S4.** **Model selection for time spent in 0 –5 m depth bin.** Selection table for generalized linear mixed models used to evaluate the relationship between the proportion of time spent in depth bins between 0 – 5 m and the monsoon period. The chosen model is bolded. Column names correspond to the following: df = degrees of freedom, AICc = Akaike information criterion corrected for sample size, ΔAICc = the difference in the AICc, wAICc = AICc weight, R^2^ Cond = the proportion of variance explained by fixed and random effects, and R^2^ Marg = the proportion of variance explained by fixed effects.

| Model (GLMM) | df | AICc | ΔAICc | wAICc | R^2^ Cond | R^2^ Marg |
| --- | --- | --- | --- | --- | --- | --- |
| 1. **PropShallow~Monsoon + (1 \| MantaID)** | **4** | **3933.575** | **0.000** | **0.999** | **0.327** | **0.075** |
| 1. PropShallow~1 + (1 \| MantaID) | 3 | 3947.107 | 13.569 | 0.001 | 0.388 | 0.000 |
