# Supplementary material for: Movement, residency, and behavioral plasticity of reef manta rays in the Samarai Islands of Papua New Guinea
Source: PLoS One. 2026 May 28;21(5):e0344615. doi: 10.1371/journal.pone.0344615 (PMC13218459; doi:10.1371/journal.pone.0344615)
Supplement: S5 Table — Selection table for generalized linear mixed models used to evaluate the relationship between the proportion of time spent in depth bins between 50–100 m and the monsoon period. The chosen model is bolded. Column names correspond to the following: df = degrees of freedom, AICc = Akaike information criterion corrected for sample size, ΔAICc = the difference in the AICc, wAICc = AICc weight, R2 Cond = the proportion of variance explained by fixed and random effects, and R2 Marg = the proportion of variance explained by fixed effects. (DOCX) [file pone.0344615.s009.docx]

**Table S5.** **Model selection for time spent in 50 – 100 m depth bin.** Selection table for generalized linear mixed models used to evaluate the relationship between the proportion of time spent in depth bins between 50 – 100 m and the monsoon period. The chosen model is bolded. Column names correspond to the following: df = degrees of freedom, AICc = Akaike information criterion corrected for sample size, ΔAICc = the difference in the AICc, wAICc = AICc weight, R^2^ Cond = the proportion of variance explained by fixed and random effects, and R^2^ Marg = the proportion of variance explained by fixed effects.

| Model (GLMM) | df | AICc | ΔAICc | wAICc | R^2^ Cond | R^2^ Marg |
| --- | --- | --- | --- | --- | --- | --- |
| 1. **PropDeep~1 + (1 \| MantaID)** | **3** | **3271.593** | **0.000** | **0.616** | **0.492** | **0.000** |
| 1. PropDeep~Monsoon + (1 \| MantaID) | 4 | 3272.536 | 0.942 | 0.384 | 0.447 | 0.006 |
