# Supplementary material for: Movement, residency, and behavioral plasticity of reef manta rays in the Samarai Islands of Papua New Guinea
Source: PLoS One. 2026 May 28;21(5):e0344615. doi: 10.1371/journal.pone.0344615 (PMC13218459; doi:10.1371/journal.pone.0344615)
Supplement: S6 Table — Selection table for generalized additive mixed models used to evaluate the relationship between the maximum diving depth of SPLASH tagged reef manta rays and the estimated monthly mixed layer depth of the region. The chosen model is bolded. Column names correspond to the following: df = degrees of freedom, AICc = Akaike information criterion corrected for sample size, ΔAICc = the difference in the AICc, wAICc = AICc weight, R2 = the proportion of variance explained by fixed and random effects. (DOCX) [file pone.0344615.s010.docx]

**Table S6. Model selection for relationship between diving depth and mixed layer depth.** Selection table for generalized additive mixed models used to evaluate the relationship between the maximum diving depth of SPLASH tagged reef manta rays and the estimated monthly mixed layer depth of the region. The chosen model is bolded. Column names correspond to the following: df = degrees of freedom, AICc = Akaike information criterion corrected for sample size, ΔAICc = the difference in the AICc, wAICc = AICc weight, R^2^ = the proportion of variance explained by fixed and random effects.

| Model (GAMM) | df | AICc | ΔAICc | wAICc | R^2^ |
| --- | --- | --- | --- | --- | --- |
| 1. **MaxDepth~s(MLD, k=4) + s(MantaID, bs=“re”)** | **10.58** | **7420.368** | **0.00** | **1** | **0.161** |
| 1. MaxDepth~1 + s(MantaID, bs=“re”) | 8.1 | 7441.579 | 21.211 | 0 | 0.140 |
